# Hospitalization expenses of coronary heart disease inpatients in China: evidence from two hospitals in Ningxia Hui autonomous region

**DOI:** 10.3389/fpubh.2024.1266456

**Published:** 2024-05-02

**Authors:** Chuanchuan Xu, Rugang Liu, Jian Wang, Stephen Nicholas

**Affiliations:** ^1^School of Humanities and Management, Ningxia Medical University, Yinchuan, China; ^2^School of Health Policy and Management, Nanjing Medical University, Nanjing, China; ^3^Center for Global Health, Nanjing Medical University, Nanjing, China; ^4^Jiangsu Provincial Institute of Health, Nanjing Medical University, Nanjing, China; ^5^School of Public Health, Nanjing Medical University, Nanjing, China; ^6^Dong Fureng Economic and Social Development School, Wuhan University, Beijing, China; ^7^Center for Health Economics and Management at School of Economics and Management, Wuhan University, Wuhan, China; ^8^Health Services Research and Workforce Innovation Centre, Newcastle Business School, University of Newcastle, Newcastle, NSW, Australia; ^9^Australian National Institute of Management and Commerce, Sydney, NSW, Australia

**Keywords:** coronary heart disease, hospitalization expenses, health policy, inpatient, cost

## Abstract

**Aim:**

The increasing morbidity from coronary health disease (CHD) has imposed a significant social and economic burden in China. We analyzed the factors affecting hospitalization expenses of CHD patients.

**Design:**

From 2012 to 2018, data on 16,726 CHD patients were collected from the hospital information system in Ningxia Hui Autonomous Region.

**Methods:**

A multiple ordered logistic regression model was used to analyze the factors affecting hospitalization expenses.

**Results:**

The average hospitalization expense was RMB30998.26 ± 29890.03. Hospital materials expenses accounted for roughly 60% of total hospitalization costs. The older adult, patients who were male, in critical health status, with longer hospital stays, unemployed, using antibiotics and undergoing an operation without incision had significantly raised hospital expenses, while those with fewer complications, no operations and self-paying for health care had reduced hospitalization costs (*p* < 0.05). The length of hospital stay played a partial mediator role (*p* < 0.05).

**Public contribution:**

Controlling the increase of medical materials costs and preventing over-consumption of hospital services by insured patients are recommended.

## Introduction

The rapid growth of health expenditures is a global issue. According to the 2019 World Health Organization (WHO) Report, health spending, which accounts for 10 percent of global gross domestic product (GDP), has been the fastest growing sector in the world economy. Controlling the rapid growth of medical expenses is also a major problem for China. China’s total health expenditure has increased fourfold in the past 10 years, from RMB1453.54 (US$209.29) billion in 2008, accounting for 4.06% of GDP (1), to RMB5799.83 (US$876.45) billion in 2018, accounting for 6.4% of GDP (2). Health expenditures have been predicted to reach RMB9447.9 (US$1345.69) billion in 2022 (3). Average hospitalization cost *per capita* was RMB9291.9 (US$1404.16) in 2018, up 4.5 percent over 2017 (2).

Coronary heart disease (CHD) is a serious heart disease caused by coronary artery atherosclerotic lesions and vascular stenosis or obstruction, resulting in myocardial ischemia, hypoxia or necrosis. With the acceleration of China’s aging population and the rising proportion of the older adult residents, the morbidity and mortality from CHD imposes an increasing social and economic burden on the hospital system, households and society (4–9). There were an estimated 11 million CHD patients in China in 2017. In terms of the causes of most deaths, CHD years of lost life (YLL) rose from the seventh place in 1990 to the second place in 2017, with the ratio rising from 1,077 YLL per 100,000 people in 1990 to 2057 YLL per 100,000 people in 2017. Between 1990 and 2017, the CHD mortality rate increased 2.53 times (10), with CHD morbidity and mortality predicted to rise dramatically in the next decade (11). To control the costs of China’s second leading source of health expenditure, this paper investigates CHD hospitalization expenses, which make up the largest proportion of the direct costs of CHD (12).

Surprisingly, there has been relatively few studies of CHD hospitalization expenditures in China. The CHD literature has investigated catastrophic medical payments faced by CHD patients and the effectiveness of the new rural cooperative medical system at alleviating the impact of CHD diseases in rural areas (13). Kelley et al. (14) measured the health care costs of the patients with heart disease in the last 5 years of life, and Huber et al. (15) examined the effect of integrated care models on medical expenditures and the quality of care in cardiovascular disease patients. Ding et al. (16) analyzed hospitalization expenditures and influencing factors for inpatients with CHD in Xi’an, China, recommending attenuating hospitalization expenditures by controlling the costs of medical consumables and shortening the length of hospital stay. Other research found that factors such as age, sex, education level, living in a higher-income community and household income had significant associations with CHD medical cost (12, 17–19). Surprisingly, other factors, such as health status on admission, complications, type of surgery, antibiotic use and admission year, likely to be correlated with CHD hospital costs have been largely neglected in the literature. To understand, and make recommendations on how to control, the rapid growth of CHD hospital expenses, we analyzed the detailed determinants of CHD hospitalization expenses, including health status on admission, complications, type of surgery, antibiotic use and admission year, as well as age, sex, education level, living in a higher-income community and household income of 16,726 CHD patients in Ningxia Hui Autonomous Region in China.

## Methods

### Data source and variables collected

Our data on 16,726 patients hospitalized for CHD from 2012 to 2018 were collected from Ningxia province’s hospital information system (HIS) of two large-scale city-based, tertiary grade iii-a, hospitals, facing the same cost structure. In China’s hierarchical medical system, Ningxia’s two tertiary hospitals had over 4,000 beds, providing advanced medical training and delivering complex healthcare for about 14%, or over 1 million, residents in Ningxia province. Only patients in the HIS diagnosed as i20-i25 in International Classifications of Disease ICD10 list were selected. The 2015 US$ to RMB average exchange rate was calculated at 6.2284. All statistical analyses were performed using Excel version 2010 and STATA version 12.0.

### Definition of variables

The dependent variable was CHD hospitalization expenses, which were divided into five quintile expenditure groups from low to high. Consistent with previous research, our independent variables comprised data on sex, age, ethnic group, marital status, occupation, type of medical insurance, admission year, health status, complications, operations, antibiotic treatment and length of hospital stay. As shown in Table 1, our sample was divided into five groups by age: <45 years old, 45–54 years old, 55–64 years old, 65–74 years old and ≥ 75 years old. Occupations mainly comprised retired (39.72%), unemployed (18.99%), farmers (16.67%) and jobholders (13.89%). As part of China’s, 2009 health reform agenda, medical insurance comprising New Rural Co-operative Medical System (NRCMI), Urban Employee Basic Medical Insurance (UEBMI) and Urban Resident Basic Medical Insurance (URBMI), covered over 95% of the population. A few people in China had no health insurance or were members of special insurance schemes, falling into the ‘other’, mainly self-paying, category. Health status means general, urgent or critical health conditions, which was determined by the physician’s evaluation of each patient’s health on admission to hospital. Complications were divided into none (0) and 1 to 5 complications. Operations were categorized as operations with incision, operations without incision (such as ultrasonic knife technology) and no operation. Antibiotics was a binary yes-no variable. Length of hospital stay was measured by the number of days of hospitalization from completing the hospitalization procedures to the discharge procedures.

**Table 1:** Characteristics of the patients.

Variables		Number	Percent	Mean of hospitalization expenses	Std.Err.	Median
Sex	Male	10,558	63.12	33471.86	299.03	20224.73					
	Female	6,168	36.88	26764.11	355.26	11730.46					
Age	<45	3,000	17.94	24172.88	490.19	11323.13					
	45–54	5,422	32.42	30800.79	407.45	14049.29					
	55–64	4,934	29.50	33152.19	437.27	19494.87					
	65–74	2,672	15.98	34363.05	587.41	30185.22					
	≥75	698	4.17	33761.40	1097.41	31771.01					
Ethnicity	Han	13,786	82.42	31175.58	256.02	14633.03					
	Hui	2,566	15.34	30194.28	568.15	14501.27					
	Others	374	2.24	29978.19	1604.55	12368.31					
Marriage	Married	16,491	98.60	31053.91	233.08	14621.17					
	Unmarried	125	0.75	30567.02	2565.68	13302.63					
	Others	110	0.66	23145.85	2237.43	10471.64					
Occupation	Retiree	6,643	39.72	28605.93	367.18	12157.34					
	Farmer	2,788	16.67	33442.30	532.77	27069.79					
	Jobholder	2,323	13.89	33578.10	655.51	18289.52					
	Self-employed	401	2.40	33771.44	1470.83	30770.67					
	Unemployed	3,176	18.99	31456.82	532.03	15370.62					
	Others	1,395	8.34	31368.80	783.97	18965.47					
Insurance	NRCMI	3,056	18.27	32527.30	515.39	21864.96					
	UEBMI	6,834	40.86	30041.13	367.55	13098.43					
	URBMI	6,645	39.73	31404.53	368.04	14764.14					
	Self-paying	191	1.14	26645.67	2072.97	10672.74					
Admission year	2012	1,605	9.60	28954.57	702.91	13980.37					
	2013	2,043	12.21	29170.12	607.64	13879.06					
	2014	2,135	12.76	30566.30	649.25	14899.24					
	2015	2,561	15.31	33014.54	646.15	14227.41					
	2016	2,689	16.08	31353.19	593.00	14962.44					
	2017	2,770	16.56	30732.15	587.51	12742.17					
	2018	2,923	17.48	31872.81	513.93	24165.3					
Health status	General	7,978	47.70	25774.06	311.94	10174.91					
	Urgent	4,415	26.40	35173.42	462.01	25052.71					
	Critical	4,333	25.91	36362.99	471.37	29751.96					
Complications	No	4,487	26.83	30267.69	272.71	14566.44					
	Yes	12,239	73.17	32991.01	433.30	17746.67					
Surgery	With incision	7,206	43.08	45068.33	338.17	43476.59					
	Without incision	2,609	15.60	51589.22	617.95	50659.59					
	No surgery	6,911	41.32	8554.23	90.68	6689.23					
Antibiotics use	No	10,664	63.76	29877.19	286.42	12438.94					
	Yes	6,062	36.24	32970.40	389.61	18361.44					

### Analysis methods

Hospitalization expenses was the dependent variable, divided into 5 groups and treated as an ordered variable. The chi-square tests of cross tables (5 × X_i_, 2 ≤ X_i_ ≤ 7) and ordered logistic regression model were used to analyze the differences in the ordered groups of medical expense by different independent variable groups. We also undertook a mediator analysis since length of hospital stay might mediate the relationship between hospital expenses and the other independent variables.

### Regression model building

We built a multiple ordered logistic regression model to analyze the relationship between hospitalization expenses and the independent variables, π_i_ = P(Y = i) (i = 1–5), where Y is ordered hospitalization expenses and π_i_ means the probability of hospitalization expense belonging to level i. The regression model can be written as Equations (1–5.


(1) $$\ln\frac{\pi_{1}}{\pi_{2}+\pi_{3}+\pi_{4}+\pi_{5}}=\alpha_{1}+X\beta \text{,}$$

(2) $$\ln\frac{\pi_{1}+\pi_{2}}{\pi_{3}+\pi_{4}+\pi_{5}}=\alpha_{2}+X\beta \text{,}$$

(3) $$\ln\frac{\pi_{1}+\pi_{2}+\pi_{3}}{\pi_{4}+\pi_{5}}=\alpha_{3}+X\beta \text{,}$$

(4) $$\ln\frac{\pi_{1}+\pi_{2}+\pi_{3}+\pi_{4}}{\pi_{5}}=\alpha_{4}+X\beta \text{,}$$

where α_j_ (j = 1–4) is a constant term and X is a vector of independent variables, and β represents the coefficients to be estimated. Odds ratio (OR) is the ratio of probability of hospitalization expense belonging to level k or below to the probability of hospitalization expense belonging to above level k. If the coefficient of X_j_ is β_j_, OR can be written as.


(5) $$\mathrm{OR}=e^{\beta_{j}}$$

## Results

### Characteristics of patients

Table 1 displays the characteristics of the 16,726 sample patients: 63.12% were male; 95.83% were aged 45–74, with an average age 64.06 ± 10.77; most patients had Han ethnicity (82.42%), were married (98.60%) and covered by UEBMI (40.86%) or URBMI (39.73%) or NRCMI (18.27%) medical insurance. Roughly two-fifths of the patients were retirees. More than half the patients’ health status was urgent or critical; on average patients had 4.11 ± 1.23 complications and about three-fifths had an operation (43.08% with incision and 15.60% without incision); one-third of patients took antibiotics; and patients stayed in hospital for 8.52 ± 4.20 days. The average hospitalization expenses was RMB30998.26 ± 29890.03 (US$4976.92 ± 4798.99). Between 2012 and 2018, Table 1 shows that the number of CHD inpatients increased gradually year-on-year. As shown in Table 2, the average CHD hospitalization expenditure far surpassed the Ningxia’s annual average *per capita* disposable income, or household income after government taxes. Hospital expenses formed 159.31% of average disposal income in 2013 falling to 112.91% in 2018, accounting for 13.5 months of the average yearly disposable income.

**Table 2:** Average CHD expense and annual *per capita* disposable income in Ningxia.

Admission year	Average expense (RMB)	Annual *per capita* disposable income (RMB)	Average expense/annual *per capita* disposable income (%)
2012	28954.57	13104.00	220.96
2013	29170.12	14566.00	200.26
2014	30566.30	15907.00	192.16
2015	33014.54	17329.00	190.56
2016	31353.19	18832.00	166.49
2017	30732.15	20562.00	149.46
2018	31872.81	22400.00	142.29

### The single factor analysis results

We divided hospital expenses into five equal percentile groups, then used the chi-square test of cross tables (5 × X_i_, 2 ≤ X_i_ ≤ 7) to analyze the relationship between the grouped ordered hospital expenses and each dependent variable. Single factor ordered logistic regression model was used to test the significance of hospital days considering it is a continuous variable. As shown in Table 3, there were significant differences (*p* < 0.05) in hospitalization expenses by age, sex, occupation, type of medical insurance, admission year, health status, complications, operations, antibiotic use and days of hospital stays. The group with the lowest hospitalization costs had the shortest length of stay (6.13 ± 2.29).

**Table 3:** Hospitalization expenses differences among subgroups.

Variables		Lowest		Lower		Middle		Higher		Highest		Chi^2^	*p*
		Number	%	Number	%	Number	%	Number	%	Number	%		
Sex	Male	1,951	18.48	1,949	18.46	1,980	18.75	2,343	22.19	2,335	22.12	222.44	**0.000**
	Female	1,395	22.62	1,396	22.63	1,365	22.13	1,002	16.25	1,010	16.37		
Age	<45	643	21.43	745	24.83	839	27.97	372	12.40	401	13.37	518.48	**0.000**
	45–54	1,004	18.52	1,134	20.91	1,187	21.89	1,007	18.57	1,090	20.10		
	55–64	995	20.17	921	18.67	828	16.78	1,091	22.11	1,099	22.27		
	65–74	572	21.41	427	15.98	387	14.48	670	25.07	616	23.05		
	≥75	132	18.91	118	16.91	104	14.90	205	29.37	139	19.91		
Ethnicity	Han	2,753	19.97	2,737	19.85	2,744	19.90	2,779	20.16	2,773	20.11	12.03	0.150
	Hui	497	19.37	534	20.81	537	20.93	498	19.41	500	19.49		
	Others	96	25.67	74	19.79	64	17.11	68	18.18	72	19.25		
Marriage	Married	3,302	20.02	3,283	19.91	3,297	19.99	3,301	20.02	3,308	20.06	14.57	0.068
	Unmarried	24	19.20	26	20.80	24	19.20	28	22.40	23	18.40		
	Others	20	18.18	36	32.73	24	21.82	16	14.55	14	12.73		
Occupation	Retiree	1,451	21.84	1,491	22.44	1,420	21.38	1,078	16.23	1,203	18.11	266.60	**0.000**
	Farmer	418	14.99	503	18.04	568	20.37	702	25.18	597	21.41		
	Jobholder	514	22.13	410	17.65	365	15.71	490	21.09	544	23.42		
	Self-employed	87	21.70	60	14.96	59	14.71	102	25.44	93	23.19		
	Unemployed	591	18.61	621	19.55	679	21.38	639	20.12	646	20.34		
	Others	285	20.43	260	18.64	254	18.21	334	23.94	262	18.78		
Insurance	NRCMI	552	18.06	526	17.21	615	20.12	726	23.76	637	20.84	82.36	**0.000**
	UEBMI	1,479	21.64	1,434	20.98	1,372	20.08	1,223	17.90	1,326	19.40		
	URBMI	1,263	19.01	1,343	20.21	1,324	19.92	1,362	20.50	1,353	20.36		
	Self-paying	52	27.23	42	21.99	34	17.80	34	17.80	29	15.18		
Admission year	2012	306	19.07	353	21.99	349	21.74	335	20.87	262	16.32	281.84	**0.000**
	2013	371	18.16	472	23.10	410	20.07	457	22.37	333	16.30		
	2014	337	15.78	464	21.73	519	24.31	431	20.19	384	17.99		
	2015	511	19.95	533	20.81	489	19.09	388	15.15	640	24.99		
	2016	567	21.09	493	18.33	578	21.49	481	17.89	570	21.20		
	2017	673	24.30	517	18.66	494	17.83	506	18.27	580	20.94		
	2018	581	19.88	513	17.55	506	17.31	747	25.56	576	19.71		
Health status	General	2,295	28.77	1,858	23.29	1,250	15.67	1,285	16.11	1,290	16.17	1000.00	**0.000**
	Urgent	533	12.07	796	18.03	1,046	23.69	1,043	23.62	997	22.58		
	Critical	518	11.95	691	15.95	1,049	24.21	1,017	23.47	1,058	24.42		
Complications	No	36	27.07	22	16.54	23	17.29	36	27.07	16	12.03	12.24	**0.016**
	Yes	3,310	19.95	3,323	20.03	3,322	20.02	3,309	19.94	3,329	20.06		
Surgery	With incision	26	0.36	909	12.61	1,532	21.26	2,535	35.18	2,204	30.59	9900.00	**0.000**
	Without incision	27	1.03	261	10.00	468	17.94	744	28.52	1,109	42.51		
	No surgery	3,293	47.65	2,175	31.47	1,345	19.46	66	0.95	32	0.46		
Antibiotics use	No	2,581	24.20	2,207	20.70	1,674	15.70	2,116	19.84	2,086	19.56	541.73	**0.000**
	Yes	765	12.62	1,138	18.77	1,671	27.57	1,229	20.27	1,259	20.77		

	Mean	Std. Dev.	Mean	Std. Dev.	Mean	Std. Dev.	Mean	Std. Dev.	Mean	Std. Dev.		
Hospital days	6.13	2.29	7.93	2.95	10.26	4.49	8.25	3.95	10.03	5.23	34.00(Z)	**0.000**

Bold value means *p* < 0.05. No bold is acceptable.

### The regression results

As shown in Table 4, the dependent variable of Model 1 and Model 3 was CHD hospitalization expenses, and the dependent variable of Model 2 was the length of hospital stay. The length of hospital stay was also added in Model 3 as a independent variable. Length of stay played a partial mediator effect (*p* < 0.05 in Model 3) between age, sex occupation, type of medical insurance, admission year, health status, complications, operations, antibiotic use and CHD hospitalization expenses (*p* < 0.05 in Model 1 and Model 2).

**Table 4:** Results of regression models.

Variables		Model 1		Model 2		Model 3		
		Coef.	*p*	Coef.	*p*	Coef.	*p*	OR
Sex	Male	(Reference group)						
	Female	−0.18	0.000	0.27	0.000	−0.27	0.000	0.76
Age	<45	(Reference group)						
	45–54	0.18	0.027	0.09	0.592	0.15	0.058	1.17
	55–64	0.22	0.005	0.20	0.225	0.18	0.026	1.20
	65–74	0.36	0.000	0.62	0.000	0.24	0.003	1.28
	≥75	0.58	0.000	1.09	0.000	0.40	0.000	1.50
Ethnicity	Han	(Reference group)						
	Hui	0.04	0.350	0.10	0.234	0.00	0.908	1.00
	Others	−0.14	0.167	−0.48	0.024	−0.05	0.625	0.95
Marriage	Married	(Reference group)						
	Unmarried	−0.28	0.104	−0.15	0.680	−0.27	0.119	0.76
	Others	−0.22	0.223	−0.61	0.114	−0.10	0.582	0.90
Occupation	Retiree	(Reference group)						
	Farmer	0.01	0.802	−0.48	0.000	0.12	0.025	1.13
	Jobholder	0.01	0.799	−0.21	0.060	0.04	0.458	1.04
	Self-employed	−0.08	0.440	−0.59	0.008	0.03	0.747	1.04
	Unemployed	0.07	0.160	−0.27	0.009	0.13	0.008	1.14
	Others	−0.10	0.119	−0.25	0.053	−0.05	0.411	0.95
Insurance	NRCMI	(Reference group)						
	UEBMI	0.43	0.003	0.75	0.013	−0.09	0.087	0.91
	URBMI	0.41	0.004	1.11	0.000	−0.06	0.165	0.94
	Self-paying	0.43	0.003	1.06	0.000	−0.31	0.035	0.73
Admission year	2012	(Reference group)						
	2013	−0.13	0.037	−1.45	0.000	0.17	0.010	1.19
	2014	0.15	0.016	−0.69	0.000	0.31	0.000	1.36
	2015	0.12	0.075	−1.11	0.000	0.34	0.000	1.41
	2016	0.04	0.536	−0.74	0.000	0.19	0.005	1.21
	2017	−0.05	0.368	−1.67	0.000	0.29	0.000	1.34
	2018	−0.72	0.000	−2.94	0.000	−0.20	0.002	0.82
Health status	General	(Reference group)						
	Urgent	0.70	0.000	0.52	0.000	0.69	0.000	1.99
	Critical	0.83	0.000	0.82	0.000	0.78	0.000	2.18
Complications	No	(Reference group)						
	Yes	0.75	0.000	1.84	0.000	0.43	0.013	1.53
Surgery	With incision	(Reference group)						
	Without incision	0.20	0.001	0.19	0.117	0.19	0.001	1.21
	No surgery	−4.06	0.000	−0.35	0.000	−4.53	0.000	0.01
Antibiotics use	No	(Reference group)						
	Yes	0.88	0.000	1.84	0.000	0.64	0.000	1.90
Hospital days						0.22	0.000	1.25

**Figure 1:**
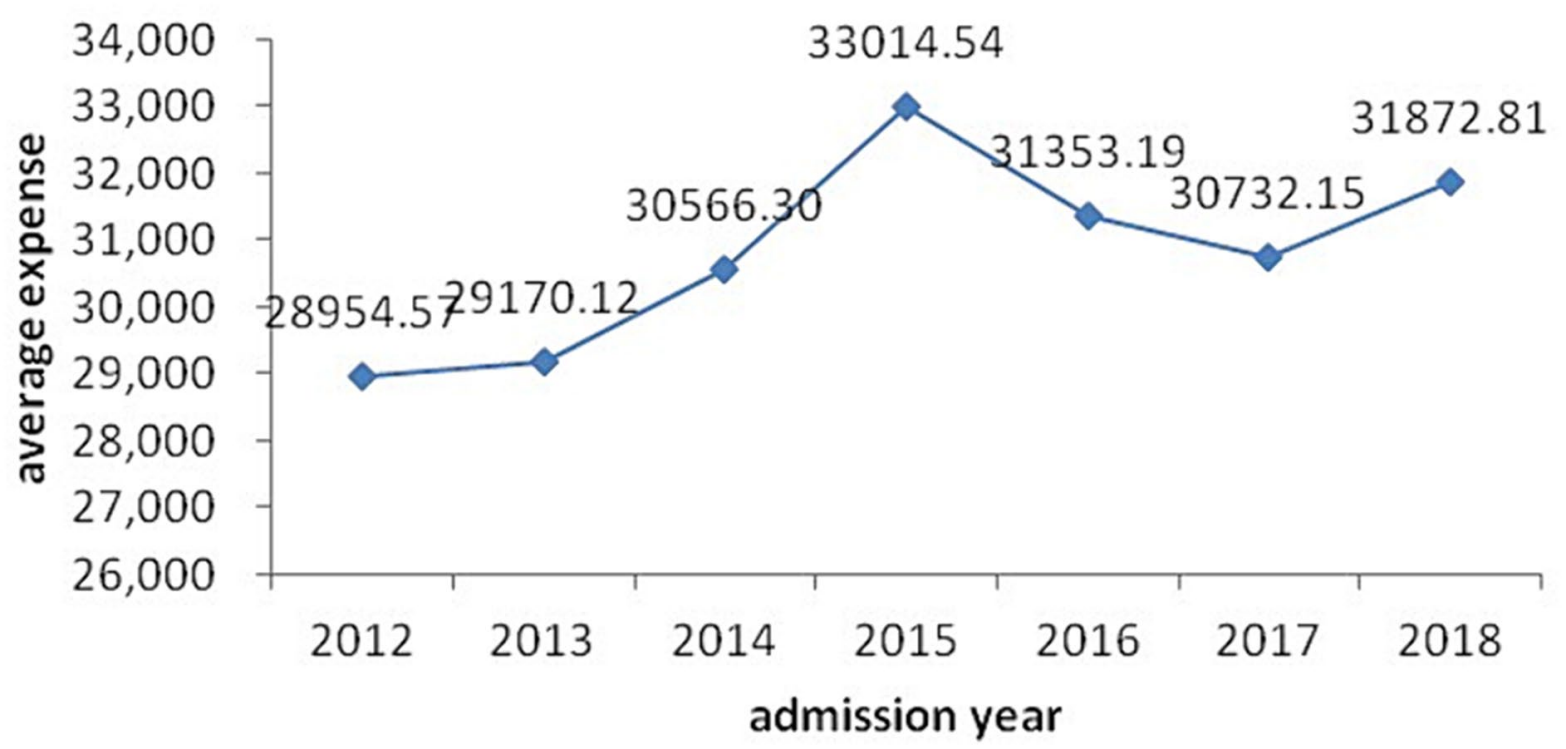
Average expense by admission year.

**Figure 2:**
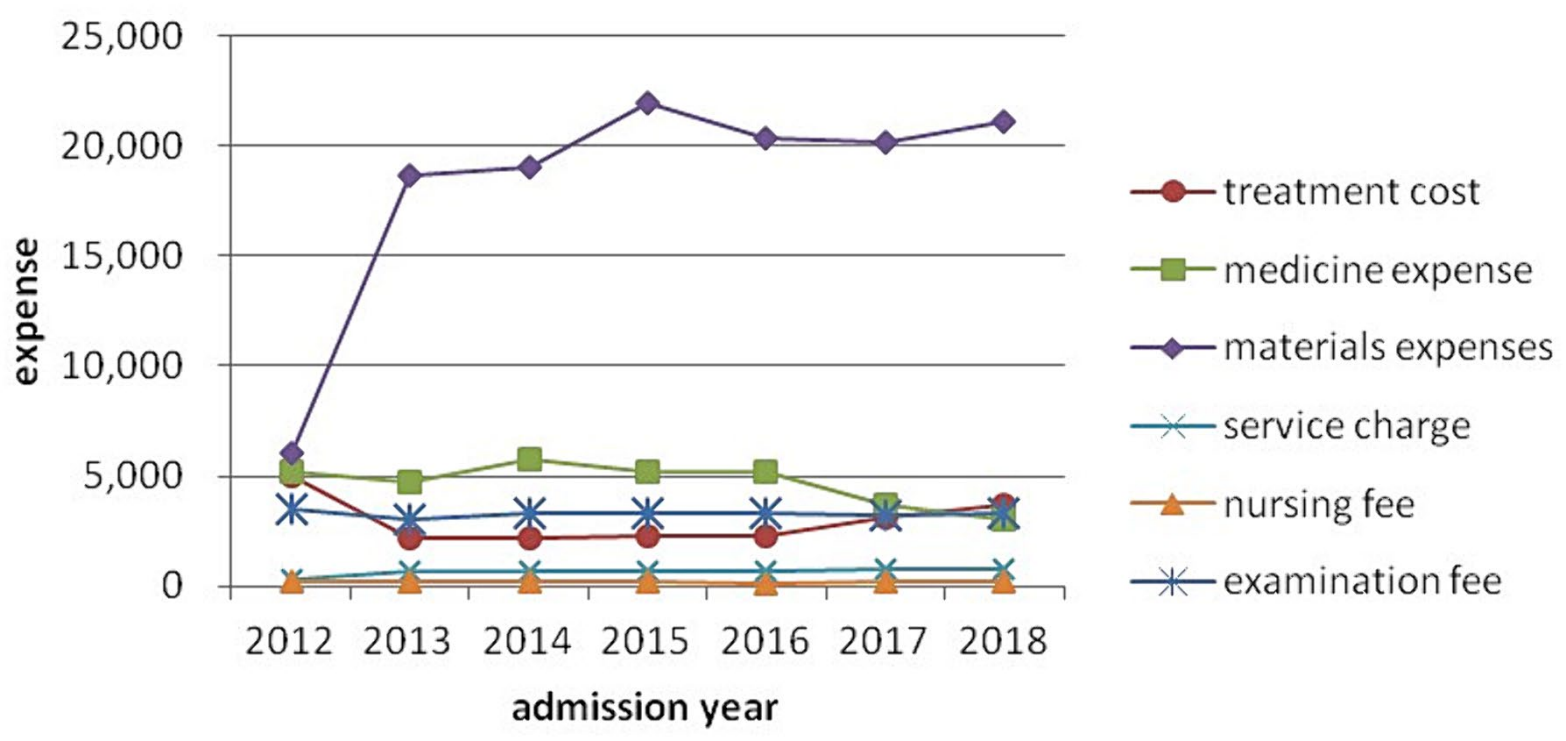
Different expenses by admission year.

Model 3 shows that age, sex occupation, type of medical insurance, admission year, health status, complications, operations, antibiotic use and length of hospital stay were significant determinants of CHD hospitalization expenses (*p* < 0.05). Table 4 shows that CHD hospitalization expenses increased with age, health situation and length of hospital stay. The expenses of patients older than 74 years had 1.5 the odds of those less than 45 years old, and those with a critical health status 2.18 the odds of those with a general health status, of being in the higher quintile. Patients who were male, unemployed, using antibiotics and undergoing an operation without incision were associated with rising hospitalization expenses, while those with few complications, no operation and self-paying expenses had reduced hospitalization costs. As shown in Figure 1, CHD hospitalization expenses rose year-on-year from 2012 to 2015 year, and then fluctuated 2016–2018, with the highest expense level occurring in 2015 at 1.41 times that in 2012. In Figure 2, we decomposed hospitalization expenses. Accounting for roughly 60% of hospitalizations costs, the proportion of materials in total hospital costs rose rapidly in 2012–2013, peaking in 2015 and remaining high until 2018. Non-materials costs remained largely constant across the study period. Nursing, service and examination expenses were mainly constant, with no increase in medical staff costs. Medicine costs showed a year-on-year downward trend from 2014 to 2018, with the most significant decline from 2016 to 2018.

## Discussion

The average direct CHD hospitalization costs were RMB30998.26 (US$4976.92) before applying for reimbursement from medical insurance. Although average CHD hospitalization costs were lower than other regions in China (20) and other countries (21), it imposed a heavy burden on CHD patients and their families in one of the least development regions in China. Given CHD hospital expenses as a proportion of the average yearly income, we recommend improving national insurance cover, catastrophe insurance and income safety nets to shield individuals and families from financial stress.

CHD hospitalization expenses varied by age and sex. Similar to other research (22), female patients were likely to incur the lower CHD expenses only 0.76 odds that of male patients. Hospitalization expenses, unsurprisingly, increased with age. Generally speaking, the older a patient the more likely he or she was to suffer CHD and other illnesses and the more heath care they accessed. Our finding that CHD Ningxia men and the older adult faced especially serious health expenses was similar to studies in Japan, Netherlands and other parts of China (23–26), which suggests that males and older adult people should be a focus of controlling CHD expenses. Whether women faced fewer CHD-related illnesses or had less access to health resources requires further research.

The CHD hospitalization expenses of the unemployed were higher than for retirees. Unemployed people constituted a vulnerable group with poor social, economic and health status. Although retirees were older in general, their overall social-economic status, health awareness and resources were higher than other occupational groups. Ethnicity was not a significant variable in hospitalization expenses.

There was no statistical difference in CHD hospitalization expenses covered by NRCMI, UEBMI and URBMI, but the expense of NRCMI patients was higher than self-pay patients. Patients covered by insurance, especially NRCMI, may be more likely to opt for expensive treatments because medical insurance covered a larger part of their medical cost, reducing their out-of-pocket hospitalization expenses. This may encourage patient over-use of hospital resources, especially those insured with more generous health insurance schemes. Also, health professionals may over-service, providing excessive hospital treatment to those who were insured or were insured on schemes with more benefits. The government needs to further evaluate and control excessive health care consumption by insured patients, assessing differential hospitalization costs by type of insurance. Similarly, noninsured patients might have received less or poorer medical care from hospitals or the uninsured might have requested less or cheaper medical care. This requires further study.

As shown in Figure 1, average hospitalization expenses increased year-on-year from 2012 to 2015, and then fluctuated during 2016–2018, with the highest average expense RMB33,014.54 (US$5,300.65) in 2015. Figure 2 reflected the effectiveness of the drug mark-up cost-control measures in China’s, 2009 national health reforms, which ended the practice of hospitals and doctors retaining a proportion of the income from drug prescriptions.

The introduction of new technologies and the application of new medical materials helps explain the increased cost of materials for CHD patients. Two major health policy changes also directly impacted materials costs. In 2012, the government increased the reimbursement ratio of medical materials in Ningxia, which might explain the rapid growth of materials expenses from 2012 to 2013. Second, the government’s ending of the drug mark-up policy in hospitals saw a decrease in the drug bill for patients, but also significantly reduced hospital income in 2014. To compensate for the loss of drug mark-up income, many hospitals increased the use of medical materials, which explains the further rise in materials expenses in 2014–2015. Developing or pursuing alternatives inexpensive medical materials is one recommendation for controlling CHD costs. Further, any drug mark-up type reforms covering medical materials should consider revising insurance schemes covering medical materials costs and should take the expected behavioral changes by patients (over-demand for medical materials), health professionals (over-providing medical materials) and hospital administrators (increasing hospital income by over-use of medical materials) into account. Measures to control hospital costs might also include open bidding and uniform procurement procedures for materials to control the large increase in the cost of materials.

Hospitalization costs are particularly sensitive to the length of the hospital stay. The dependent variables including age, sex occupation, type of medical insurance, admission year, health status, complications, operations and antibiotic use influenced CHD hospitalization expenses directly and also indirectly through the length of the hospital stay. Compared to insured patients, those without insurance had significantly shorter hospital stays, which may have reflected patient choice. For insured patients, one possible reason for longer hospital stays was patients’ health conditions became more severe and need more treatment. But insurance policy changes may have influenced the length of hospital stay. The 2009 health reforms saw changes to the health insurance benefit schedules, creating differential reimbursements and out-of-pocket expenses across insurance schemes. For those on more generous reimbursement insurance schemes, physicians may have deliberately prolonged hospital stays to increase hospital income, especially when the end of the drug mark-ups negatively impacted hospital finances. Subject to ensuring the quality of health care, we recommend shorting any unnecessary length of hospital stay and speeding up bed turnover rates, which would improve the effective utilization of health resources, control medical expenses and improve hospital efficiency. Government should also consider standardizing the benefit schemes across insurance schemes to reduce both over-treatment by doctors and over-demand of hospital services by patients. On the reverse side, some hospitals forced patients to be discharged or transferred to another hospital who had not fully recovered in order to reduce their average length of patient stay records. Discharging patients prematurely to reduce length of stay statistics pose significant dangers to patients’ health and well-being.

Those classified as having a ‘serious’ health status when first hospitalized, undergoing operations and using antibiotics, were likely sicker and requiring more treatment than other patients, thus incurring higher hospitalization expenses. Another possible reason for higher hospitalization costs among patients using antibiotics was the prevalence of infections in hospitals. Studies have shown that hospitalization costs for patients with infections in hospital were significantly higher than those without infections. Control of infection rates in hospital reduce the use of antibiotics, decreasing the cost of treating CHD hospitalized patients. The advantages of non-invasive surgery include reduced trauma, shorter operation time and quicker postoperative recovery (27–29). However, the expenses of non-invasive surgery for CHD patients were significantly higher than that for ordinary surgery. We recommend a cost-effectiveness analysis to determine whether CHD non-invasive or ordinary surgery are better for patients and cost effective for the health system.

### Limitations

Although 16,726 patients covering 14% of Ningxia catchment area were involved in our study, data were drawn from only two of the largest hospitals in Ningxia, which may lead to selection bias. Larger numbers and types of hospitals, such as lower level county, district and township hospitals or village and primary health facilities, should be included in future studies and also studies of hospitals in other provinces. The findings may not be representative of higher level tertiary hospitals in other provinces, which can be confirmed by comparative studies in other regions. The HIS did not include data on education, and our data and conclusions apply to costs outcomes, and future studies should discuss non-cost outcomes.

## Conclusion

Patient age, sex, occupation, type of medical insurance, admission year, health situation, complications, operations, antibiotic use and length of hospital stay all contributed to CHD hospitalization expenses. The length of hospital stay was a partial mediator between age, sex occupation, type of medical insurance, admission year, health status, complications, operations, antibiotic use and CHD hospitalization expenses. The hospitalization expenses of male, older and unemployed CHD patients were significantly higher than other patients. Future study should focus on these subpopulations. Material expenses accounted for the majority of CHD hospitalization costs. It is necessary to review hospitalization costs, especially materials use, related to insured patients, which were significantly higher than self-paying patients. Policies such as raising the reimbursement ratio of medical materials (2012–2013) and abolishing the drug mark-up in hospitals (2014–2015) contributed to an increase in materials expenses. New health policies need to balance the interests of all parties, including hospitals whose financing can be adversely affected by policy changes. Controlling infections in hospital, shortening hospitalization time and choosing appropriate surgical methods will help control hospitalization cost of CHD patients.

## Data availability statement

The raw data supporting the conclusions of this article will be made available by the authors, without undue reservation.

## Ethics statement

Since all data were collected from the HIS anonymized database that did not contain any personal information, such as nameormedical record number,ethics approval was not required.

## Author contributions

CX: Conceptualization, Data curation, Formal analysis, Investigation, Methodology, Project administration, Resources, Software, Supervision, Validation, Visualization, Writing – original draft, Writing – review & editing. JW: Conceptualization, Data curation, Methodology, Resources, Visualization, Writing – review & editing. SN: Conceptualization, Funding acquisition, Resources, Supervision, Visualization, Writing – review & editing. RL: Conceptualization, Data curation, Formal analysis, Funding acquisition, Methodology, Project administration, Resources, Software, Supervision, Validation, Visualization, Writing – original draft, Writing – review & editing.
